# Efficacy of a Moderately Low Carbohydrate Diet in a 36-Month Observational Study of Japanese Patients with Type 2 Diabetes

**DOI:** 10.3390/nu10050528

**Published:** 2018-04-24

**Authors:** Mariko Sanada, Chinatsu Kabe, Hisa Hata, Junichi Uchida, Gaku Inoue, Yoko Tsukamoto, Yoshifumi Yamada, Junichiro Irie, Shogo Tabata, Mitsuhisa Tabata, Satoru Yamada

**Affiliations:** 1 Kitasato Institute Hospital, Diabetes Center, 5-9-1 Shirokane, Minato-ku, Tokyo 108-0072, Japan; mrksmd@insti.kitasato-u.ac.jp (M.S.); kabe@insti.kitasato-u.ac.jp (C.K.); h-izumi@insti.kitasato-u.ac.jp (H.H.); uchida-j@insti.kitasato-u.ac.jp (J.U.); inoueg@pharm.kitasato-u.ac.jp (G.I.); tsuka-y@insti.kitasato-u.ac.jp (Y.T.); sky_walker_718@a6.keio.jp (S.T.); mitsutabata@gmail.com (M.T.); 2 Boei Ika Daigakko Boei Igagku Kenkyu Center, 3-2 Namiki, Tokorozawa, Saitama 359-0042, Japan; yamayoshi0718@yahoo.co.jp; 3 School of Medicine, Keio University, Internal Medicine, 35 Shinanomachi, Shinjuku, Tokyo 160-8582, Japan; j-irie@z8.keio.jp

**Keywords:** low-carbohydrate diet, type 2 diabetes mellitus, observational study

## Abstract

We previously showed that a non-calorie-restricted, moderately low-carbohydrate diet (mLCD) is more effective than caloric restriction for glycemic and lipid profile control in patients with type 2 diabetes. To determine whether mLCD intervention is sustainable, effective, and safe over a long period, we performed a 36-month observational study. We sequentially enrolled 200 patients with type 2 diabetes and taught them how to follow the mLCD. We compared the following parameters pre- and post-dietary intervention in an outpatient setting: glycated hemoglobin (HbA1c), body weight, lipid profile (total cholesterol, low and high-density lipoprotein cholesterol, triglycerides), systolic and diastolic blood pressure, liver enzymes (aspartate aminotransferase, alanine aminotransferase), and renal function (urea nitrogen, creatinine, estimated glomerular filtration rate). Data from 157 participants were analyzed (43 were lost to follow-up). The following parameters decreased over the period of study: HbA1c (from 8.0 ± 1.5% to 7.5 ± 1.3%, *p* < 0.0001) and alanine aminotransferase (from 29.9 ± 23.6 to 26.2 ± 18.4 IL/L, *p* = 0.009). Parameters that increased were high-density lipoprotein cholesterol (from 58.9 ± 15.9 to 61.2 ± 17.4 mg/dL, *p* = 0.001) and urea nitrogen (from 15.9 ± 5.2 to 17.0 ± 5.4 mg/dL, *p* = 0.003). Over 36 months, the mLCD intervention showed sustained effectiveness (without safety concerns) in improving HbA1c, lipid profile, and liver enzymes in Japanese patients with type 2 diabetes.

## 1. Introduction

The most recent dietary guidelines of the American Diabetes Association (ADA) emphasize that no single diet is suitable for all people with diabetes, and likewise there is no ideal macronutrient balance [1]. Several dietary approaches have been proposed in Western countries for people with diabetes [1,2], with guidelines recommending that individuals discuss with health professionals (physicians and dietitians) which approach would be preferable, most effective, and sustainable for them. 

Recently, the Japanese Diabetes Society relaxed their dietary approach, recommending a caloric restriction of 25–35 kcal/kg ideal body weight with carbohydrates composing 50–60% of total energy consumption. No other dietary approach is currently approved by the Japanese Diabetes Society guidelines [3]. In an endeavor to make dietary approaches more flexible and sustainable for diabetes patients in Japan, in August 2009 we adopted a non-calorie restricted, moderately low-carbohydrate diet (mLCD) with the approval of the Institutional Ethical Review Board of our hospital in Tokyo, Japan. In a randomized clinical trial, we found this mLCD to be more effective than caloric restriction for glycemic and lipid profile control in patients with type 2 diabetes [4]. However, this trial was limited to six months in duration. To evaluate the long-term efficacy and safety of mLCD as a sustainable dietary therapy and to check for rebound effects (which are common in dietary studies [5]), we conducted a 36-month observational study of patients with diabetes following our mLCD.

## 2. Materials and Methods

We retrospectively enrolled 200 patients with type 2 diabetes who received outpatient treatment in Kitasato Institute Hospital, Tokyo, Japan between August 2009 and October 2016. Participants were instructed to restrict their carbohydrate intake to 20–40 g per meal and 70–130 g per day, with 10 g of carbohydrates consumed as a snack or drink at least once daily. Although we did not recommend any percentage of carbohydrate, fat, and protein to total caloric intake, it was 30:45:20 in our previous study [4]. A dietary salt restriction intervention was also performed on patients for whom it was deemed appropriate. Patients who injected insulin were recommended to monitor their capillary glucose levels frequently after mLCD was initiated. 

At the first nutritional intervention and at six-month intervals thereafter, we measured the following: glycated hemoglobin (HbA1c), body weight, lipid profile (total cholesterol (TC), low-density lipoprotein cholesterol (LDL-C), high-density lipoprotein cholesterol (HDL-C), triglyceride (TG)), blood pressure (systolic blood pressure (SBP), diastolic blood pressure (DBP)), liver enzymes (aspartate aminotransferase (AST), alanine aminotransferase (ALT)), and renal function (urea nitrogen (UN), uric acid (UA), creatinine (Cr), estimated glomerular filtration rate (eGFR)). We also recorded the incidence of hypoglycemia (defined as self-monitored blood glucose levels less than 70 mg/dL, with or without hypoglycemic symptoms) for 2 months before the first intervention and for 2-month intervals during the intervention, and calculated the corresponding before/after ratios. Missing values were replaced with values obtained during the previous or following two months.

To investigate the interactions between response to the mLCD and participant baseline HbA1c level or body mass index (BMI, kg/m^2^), we conducted post hoc analyses. In one analysis, we classified participants according to their change in HbA1c during the 36 months of dietary intervention as follows: responder (decrease in HbA1c), unchanged (no change in HbA1c), or worsened (increase in HbA1c). In other analyses, we classified participants according to their baseline HbA1c (HbA1c < 7%, 7% ≤ HbA1c < 8%, 8% ≤ HbA1c < 9%, or 9% ≤ HbA1c) or BMI (BMI < 25, 25 ≤ BMI < 30, or 30 ≤ BMI).

Values are presented as the mean ± standard deviation (SD). Statistical analyses were performed using IBM SPSS 19 software (IBM Japan, Tokyo, Japan). We compared baseline and 36-month parameter values using the Wilcoxon signed-rank test. Multiple comparisons were made as appropriate using two-way analysis of variance (ANOVA).

This study was approved by the institutional review board of Kitasato Institution Hospital and performed in accordance with the Declaration of Helsinki. Informed consent was obtained in the form of an opt-out, by posting notices requesting that diabetes patients who had received guidance about low carbohydrate diets inform us if they did not want their records to be used for research purposes. This consent process was approved by the ethical review board at Kitasato Institution Hospital. All participants’ anonymity is preserved. This study was registered as clinical trial ID UMIN000022910.

## 3. Results

The characteristics of the 200 patients enrolled are shown in Table 1. Of these, 43 participants were lost to follow-up during the 36-month study period. The most common reasons for drop-out were a discontinuation of visits to our hospital (*n* = 24) and referral to general physicians (*n* = 15). The other four participants lost to follow-up died (two from myocardial infarction, one from cardiac arrest, and one from a head injury). There were no differences in the baseline characteristics between the 43 patients who dropped out and the remaining 157.

**Figure 1:**
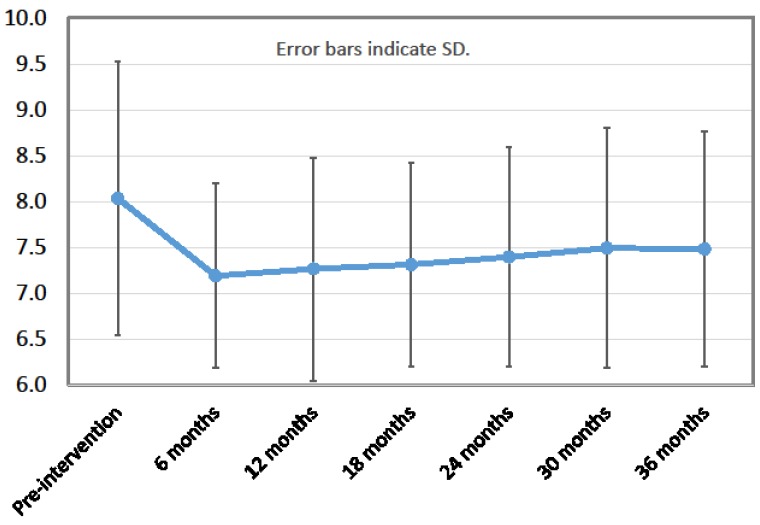
HbA1c level (%). Error bars indicate standard deviation (*n* = 157).

**Figure 2:**
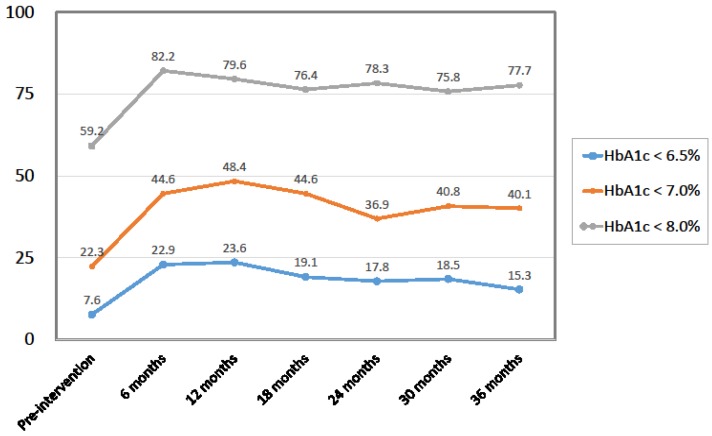
Percentage of patients who reached target HbA1c levels (%).

Among the 157 patients, HbA1c levels had improved (decreased) in 104 participants (i.e., the responder rate was 66.2%), remained unchanged in 10 participants, and worsened (increased) in 43 patients at 36 months after first mLCD intervention. From baseline to 36 months, there were statistically significant improvements in HbA1c (decrease), DBP (decrease), TC (decrease), LDL-C (increase), and ALT (decrease) (Table 2). For HbA1c levels, the improvement occurred during the first 6 months and was maintained through 36 months (Figure 1 and Figure 2). The UN level increased significantly over 36 months, while Cr and eGFR showed no change. No significant changes were observed during the study period for body weight, HDL-C, TG, SBP, AST, or UA (Table 2). The record of hypoglycemic agents showed that the number of participants taking insulin injections and/or sulfonylureas did not change during the study period. As for dosage, the mean total daily dose of insulin per person decreased from 29.9 units at the start of the intervention to 23.6 units at 36 months post-intervention. The mean dose of glimepiride (which all sulfonylurea users were prescribed) per person also decreased, from 1.39 mg at the start of the intervention to 1.11 mg at 36 months. Participants who reduced their insulin and/or glimepiride dosage started taking metformin and/or dipeptidyl peptidase-4 inhibitors instead. Thus, the number of participants taking metformin and dipeptidyl peptidase-4 inhibitors increased during the 36 months (from 71 to 89 and 48 to 93, respectively).

FBS, fasting blood sugar; SBP, systolic blood pressure; DBP, diastolic blood pressure; HbA1c, glycated hemoglobin; TC, total cholesterol; LDL-C, low-density lipoprotein cholesterol; HDL-C, high-density lipoprotein cholesterol; TG, triglyceride; LOCF, last-observation carried forward; AST, aspartate aminotransferase; ALT, alanine aminotransferase; Cr, creatinine; eGFR, estimated glomerular filtration rate; UA, uric acid; UN, urea nitrogen; ACR, albumin to creatinine ratio (from random spot urine sampling); n.s., not significant.

During the 36 months, there was also an increase in the prescription of anti-hyperlipidemic agents. Thus, we performed a last-observation carried forward (LOCF) analysis on lipid profiles using the values when anti-hyperlipidemic agents were started or when their dosages increased. This analysis showed that LOCF TC improved, and LOCF TG worsened (Table 2).

Of the 43 participants receiving insulin, 31 maintained blood glucose self-monitoring records. Among these 31 participants, the incidence of hypoglycemia was 1.46% before the introduction of mLCD, which then increased to a maximum of 2.43% for 1–2 months after the first intervention. Several months later, the incidence of hypoglycemia stabilized at approximately 1.0% (Table 3).

**Figure 3:**
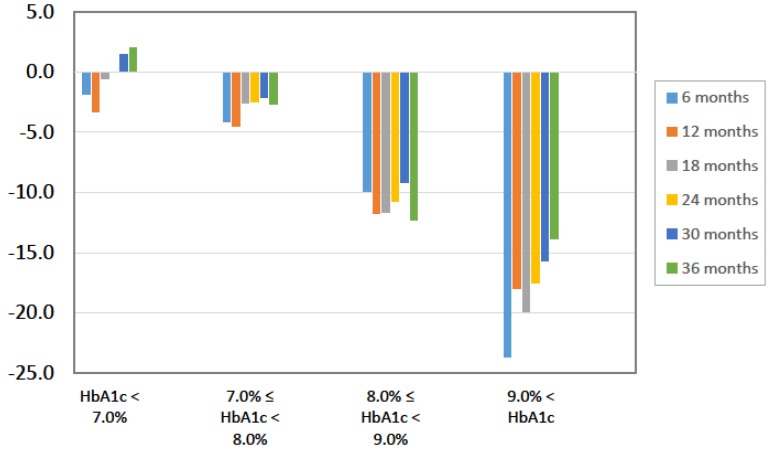
Percent changes in HbA1c stratified by baseline HbA1c (%).

To identify characteristics of participants who responded to the mLCD, we classified the 157 participants according to their change in HbA1c levels from baseline to 36 months as either responders, unchanged non-responders, or worsened non-responders. At baseline, the responders were younger and had higher HbA1c levels than the other two groups (Table 4). We also classified participants according to their baseline HbA1c levels and found that the HbA1c ≥ 9% group had the greatest improvement in HbA1c (Figure 3). On the other hand, the ALT ≥ 9% group had the greatest improvement in ALT. Change in body weight was independent of baseline HbA1c.

**Figure 4:**
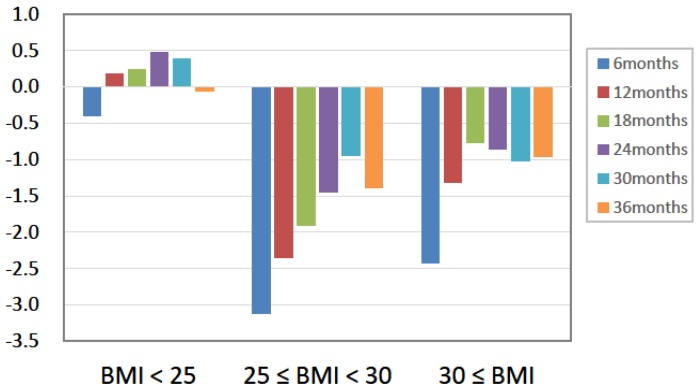
Absolute changes in body weight stratified by baseline BMI (kg).

We then classified the participants according to baseline BMI. While HbA1c decreased independently of baseline BMI level, the change in body weight across the study period differed between BMI groups. While participants with a baseline BMI < 25 showed sustained body weight, and those with BMI ≥ 25 showed a decrease in body weight (Figure 4).

Although two participants experienced myocardial infarction and died, there was no incidence of stroke, peripheral artery disease, renal dysfunction, or liver dysfunction among the participants in this study. The two patients who died from myocardial infarction had a 10+ year history of diabetes and presented multiple risk factors including hypertension, type IIb dyslipidemia, obesity, and a history of smoking.

## 4. Discussion

This study has three noteworthy aspects. First, this study is a long-term (36 months) observational study on a dietary approach for diabetes. To the best of our knowledge, this is the first long-term observational study on such a diet in East Asians. This moderately low-carbohydrate diet improved HbA1c levels within the first 6 months and then maintained that improvement for 36 months. According to the recently published ADA Standards of Medical Care [6], the effects of low-carbohydrate diets remain unclear because any improvements by such a diet tend to occur on a short-term basis and are not maintained. As described by the ADA, the wide range of definitions for a low-carbohydrate diet have created confusion. According to our current data, the improvements caused by a moderately low-carbohydrate diet can be maintained. Furthermore, the fact that East Asians have traditionally consumed a high-carbohydrate diet may explain the finding that a moderately low-carbohydrate diet is effective in reducing HbA1c in this population. 

Second, we found that a moderately low-carbohydrate diet improved HbA1c levels without causing undernutrition in non-obese patients with diabetes. Previous dietary approaches for diabetes have been considered successful if they result in weight loss. While this may be acceptable for white and black patients with type 2 diabetes, almost all of whom are obese [7,8], such diets might cause undernutrition in East Asians who develop diabetes in the absence of obesity [9]. In fact, there have been almost no studies of East Asians consuming a Mediterranean diet or the DASH diet. The CALERIE trial indicated that caloric restrictions cause loss of muscle and bone mineral density in non-obese individuals [10,11]. Thus, a moderately low-carbohydrate diet might be a safe dietary approach for East Asians or diabetics who are not obese. 

Third, our findings indicate the safety of a moderately low-carbohydrate diet. Diminished kidney function and a worse lipid profile are common concerns with a restricted carbohydrate diet [12]. In the current study, there were statistically significant changes in kidney function (eGFR; from 74.4 ± 18.8 to 72.1 ± 20.8 (mL/(min·1.73 m^2^)) during 36 months) and lipid profiles, but those changes were either not clinically significant. As for kidney function it may represent improvements compared with previous data, which have shown an annual eGFR decline of 0.7–2.0 mL/(min·1.73 m^2^) in diabetic patients [13,14]. In addition, the frequency of hypoglycemia increased in our patients using a sulfonylurea or insulin, but those patients recovered once the dose of that medication was adjusted. We therefore conclude that a moderately low-carbohydrate diet is a highly safe diet.

As for low-carbohydrate diets, there is a controversy whether high fat or high protein is more beneficial. Most of previous studies of low-carbohydrate diets were high fat and Feinman et al. described high fat as being recommended in general [15]. However, meta-analysis of Clifton et al. showed the importance of protein [16]. Our current study did not provide any information to this controversy.

This study has several limitations. Because this was an observational study and it did not compare subjects to a control group, bias, and confounding factors cannot be ruled out. In addition, the sample size of 200 patients precluded any examination of the effects of the low-carbohydrate diet on cancer or dementia. 

In conclusion, we have shown that a moderately low-carbohydrate diet is highly effective, safe, and sustainable. To address the study’s limitations, large-scale long-term randomized controlled trials should be conducted in the future.

## Figures and Tables

**Table 1:** Baseline characteristics of the study participants

	All Participants	Retained	Lost to Follow-Up
*n*	200	157	43
Female/Male	71/129	51/106	20/23
Age	59.7 ± 12.9	59.5 ± 12.4	60.5 ± 13.9
BMI (kg/m^2^)	26.4 ± 4.9	26.6 ± 4.7	25.4 ± 5.1
FPG (mg/dL)	151 ± 57	153 ± 58	145 ± 50
HbA1c (%)	8.0 ± 1.5	8.0 ± 1.5	8.0 ± 1.5
TG (mg/dL)	147 ± 120	147 ± 121	144 ± 106
LDL-C (mg/dL)	116 ± 33	116 ± 33	121 ± 45
HDL-C (mg/dL)	60 ± 17	59 ± 16	62 ± 21
BP (mmHg)	128 ± 15/76 ± 12	128 ± 15/77 ± 12	127 ± 16/72 ± 12

Values are the mean ± standard deviation. BMI, body mass index; FPG, fasting plasma glucose; HbA1c, glycated hemoglobin; TG, triglyceride; LDL-C, low-density lipoprotein cholesterol; HDL-C, high-density lipoprotein cholesterol; BP, blood pressure.

**Table 2:** Changes in the outcome measures of study participants on a moderately low-carbohydrate diet over 36 months.

	Pre-Intervention		12 Months	24 Months	36 Months	*p* Value
Body weight (kg)	72.5 ± 15.2		71.6 ± 14.9	72.0 ± 15.0	71.9 ± 15.1	n.s.
FBS (mg)	153.1 ± 58.0		143.3 ± 47.2	144.4 ± 48.6	140.6 ± 43.5	n.s.
SBP (mmHg)	127.7 ± 15.4		125.8 ± 13.3	127.1 ± 13.1	125.0 ± 13.1	n.s.
DBP (mmHg)	76.8 ± 11.8		74.8 ± 10.9	75.4 ± 9.5	74.5 ± 10.6	0.029
HbA1c (%)	8.0 ± 1.5		7.3 ± 1.2	7.4 ± 1.2	7.5 ± 1.3	<0.0001
TC (mg/dL)	200.7 ± 44.1		194.0 ± 39.0	192.8 ± 32.9	189.9 ± 33.3	0.003
LDL-C (mg/dL)	116.1 ± 33.0		107.2 ± 28.2	108.1 ± 26.7	106.7 ± 26.9	<0.0001
HDL-C (mg/dL)	59.0 ± 15.9		61.7 ± 16.7	61.2 ± 16.9	59.8 ± 18.3	n.s.
TG (mg/dL)	146.6 ± 120.7		142.7 ± 137.4	141.2 ± 102.9	152.5 ± 122.2	n.s.
LOCF TC (mg/dL)	200.8 ± 44.1		196.2 ± 40.3	196.2 ± 35.9	189.9 ± 33.3	0.0007
LOCF LDL-C (mg/dL)	116.1 ± 33.0		109.0 ± 29.7	110.6 ± 29.7	106.7 ± 26.9	n.s.
LOCF HDL-C (mg/dL)	59.1 ± 15.9		61.4 ± 16.4	61.3 ± 16.7	59.8 ± 18.3	n.s.
LOCF TG (mg/dL)	146.5 ± 120.7		147.7 ± 139.6	144.7 ± 113.6	152.5 ± 122.2	0.02
AST (IU/L)	26.6 ± 13.3		24.2 ± 10.8	25.1 ± 11.7	26.4 ± 13.6	n.s.
ALT (IU/L)	29.9 ± 23.6		23.4 ± 15.3	25.1 ± 18.5	26.2 ± 18.4	0.009
Cr (mg/dL)	0.8 ± 0.2		0.8 ± 0.2	0.8 ± 0.2	0.8 ± 0.2	0.046
eGFR (mL/(min·1.73 m^2^))	74.4 ± 18.8		73.6 ± 19.2	73.1 ± 19.0	72.1 ± 20.8	0.007
UA (mg/dL)	5.8 ± 1.4		5.9 ± 1.4	5.9 ± 1.4	6.0 ± 1.7	n.s.
UN (mg/dL)	15.8 ± 5.0		17.5 ± 6.2	16.8 ± 5.0	17.0 ± 5.5	0.002
ACR (mg/g Cr)	196.6 ± 828.0		123.6 ± 517.1	166.4 ± 515.1	123.3 ± 287.4	n.s.
Urinary protein	(−)*	91 (58%)	102 (65%)	92 (59%)	99 (63%)	n.s. †
	(+/−)*	38 (24%)	38 (24%)	40 (25%)	31 (20%)	
	(1+) *	20 (13%)	14 (9%)	17 (11%)	23 (15%)	
	(2+) *	8 (5%)	3 (2%)	8 (5%)	4 (2%)	

† Result of a chi-square test. Values are the mean ± standard deviation or number (percentage) of participants. * Although urinary protein test is qualitative, these content were similar with below; (−) 10 mg/dL, (+/−) 10–20 mg/dL, (1+) 100 mg/dL, (2+) 300 mg/dL

**Table 3:** Incidence of hypoglycemia in the 31 participants who received insulin and maintained blood glucose self-monitoring records while on a moderately low-carbohydrate diet for 36 months.

2-Month Pre-Intervention Period	1 to 2 Months	3 to 4 Months	5 to 6 Months	7 to 8 Months	9 to 10 Months	11 to 12 Months
31/2121 (1.46%)	73/3010 (2.43%)	60/2887 (2.08%)	39/2387 (1.63%)	49/2709 (1.81%)	53/2367 (2.24%)	17/2098 (0.81%)
	13 to 14 months	15 to 16 months	17 to 18 months	19 to 20 months	21 to 22 months	23 to 24 months
	44/2698 (1.63%)	26/2634 (0.99%)	26/2544 (1.02%)	12/2347 (0.51%)	30/2430 (1.23%)	20/2562 (0.78%)
	25 to 26 months	27 to 28 months	29 to 30 months	31 to 32 months	33 to 34 months	35 to 36 months
	19/2361 (0.80%)	26/2657 (0.98%)	23/2609 (0.88%)	33/2711 (1.22%)	44/2748 (1.60%)	31/3066 (1.01%)

Values are the number of hypoglycemic readings/total number of readings (percent incidence of hypoglycemia).

**Table 4:** Baseline characteristics of participants classified as responders or non-responders according to their change in HbA1c levels over the 36-month study period.

Characteristic	All (*n* = 157)	Responders (*n* = 109)	Non-Responders	
			Worsened (*n* = 41)	Unchanged (*n* = 7)
Age (years)	59.5	57.7	63.8	62.6
BMI (kg/m^2^)	26.6	26.9	26.2	25.3
FPG (mg/dL)	153	159	144	116
HbA1c (%)	8.0	8.3	7.4	7.0
TG (mg/dL)	147	159	119	112
LDL-C (mg/dL)	116	118	112	115
HDL-C (mg/dL)	59.0	56.9	61.8	76.6
BP (mmHg)	128/77	128/78	128/74	122/72
Dietary education sessions	2.2	2.3	2.2	2.1
Disease duration (years)	10.0	9.7	10.6	11.3

Values are the means. BMI, body mass index; FPG, fasting plasma glucose; HbA1c, glycated hemoglobin; TG, triglyceride; LDL-C, low-density lipoprotein cholesterol; HDL-C, high-density lipoprotein cholesterol; BP, blood pressure.
